# Distributed Systematic Network Coding for Reliable Content Uploading in Wireless Multimedia Sensor Networks

**DOI:** 10.3390/s18061824

**Published:** 2018-06-05

**Authors:** Phuc Chau, Jitae Shin, Jaehoon (Paul) Jeong

**Affiliations:** 1 School of Electronic and Electrical Engineering, Sungkyunkwan University, Suwon 16419, Korea; cmphuc@skku.edu; 2 Department of Interaction Science, Sungkyunkwan University, Suwon 16419, Korea; pauljeong@skku.edu

**Keywords:** wireless multimedia sensor networks, network coding, multimedia uploading, low-latency communication, Internet of Things

## Abstract

Recently, the wireless sensor network paradigm is shifting toward research aimed at enabling the robust delivery of multimedia content. A challenge is to deliver multimedia content with predefined levels of Quality of Service (QoS) under resource constraints such as bandwidth, energy, and delay. In this paper, we propose a distributed systematic network coding (DSNC) scheme for reliable multimedia content uploading over wireless multimedia sensor networks, in which a large number of multimedia sensor nodes upload their own content to a sink through a cluster head node. The design objective is to increase the reliability and bandwidth-efficient utilization in uploading with low decoding complexity. The proposed scheme consists of two phases: in the first phase, each sensor node distributedly encodes the content into systematic network coding packets and transmits them to the cluster head; then in the second phase, the cluster head encodes all successfully decoded incoming packets from multiple sensor nodes into innovative systematic network coding packets and transmits them to the sink. A bandwidth-efficient and channel-aware error control algorithm is proposed to enhance the bandwidth-efficient utilization by dynamically determining the optimal number of innovative coded packets. For performance analysis and evaluation, we firstly derive the closed-form equations of decoding probability to validate the effectiveness of the proposed uploading scheme. Furthermore, we perform various simulations along with a discussion in terms of three performance metrics: decoding probability, redundancy, and image quality measurement. The analytical and experimental results demonstrate that the performance of our proposed DSNC outperforms the existing uploading schemes.

## 1. Introduction

Wireless multimedia sensor networks (WMSNs) have attracted many researchers due to potential applications. Apart from environmental monitoring, WMSNs can enable some new applications such as object recognition, tracking, multimedia surveillance, automated assistance for elderly and family monitoring, and industrial process control, etc. [1]. As with sensor networks, WMSNs are composed of wirelessly connected devices that can collect information from environments at any time. However, the types of collected information are video, audio streams, images, and scalar sensor data. To enable the above practical applications, WMSNs require sensors to upload collected information to users or applications. In general, the multimedia sensors are densely distributed and divided into several clusters. Each of them consists of a single leader (i.e., cluster head (CH) node) and several ordinary nodes (ONs). A cluster head operates as a relay between its members (i.e., ONs) and base station (i.e., sink in WMSNs) [2]. The operation of data collection consists of two phases: first, each ON uploads its data packets to the corresponding CH, and second, the CH transmits the gathered data to the sink.

To enhance the efficient uploading in WMSNs, many works have paid attention to techniques such as data aggregation [2], compressive sensing [3], information fusion [4], network lifetime [5], and energy efficiency [6,7,8,9,10]. Instead of uploading raw data, CH tries to reduce the gathered information used for uploading along with guaranteeing quality of service (QoS). Those methods can increase the energy-efficient and bandwidth-efficient utilization. Nevertheless, a safe solution to satisfy all users is to upload all of the raw data regardless of whether or not the information is needed by the users. Hence, in this paper, we propose a distributed systematic network coding (DSNC) scheme for multimedia content uploading over WMSNs to increase the uploading efficiency in terms of reliability and bandwidth-efficient utilization with low decoding complexity. The uploading mechanism consists of two phases: (1) multiple ONs encode original packets into systematic network coding (SNC) packets, and transmit them to the dedicated CH using a proposed bandwidth-efficient and channel-aware error control algorithm in the first phase, where each node uses a different frequency [11]); (2) instead of conventionally forwarding all the received SNC packets from multiple senders to the sink, the CH decodes the SNC packets and encodes them into innovative SNC packets in the second phase. Particularly, our contributions are listed as follows:

- The proposed DSNC enables to enhance the efficient multimedia content uploading and to solve the issue of heavy feedback signaling and retransmission caused by retransmission-based protocols in WMSNs.
- We propose a bandwidth-efficient and channel-aware error control algorithm to enhance the bandwidth-efficient utilization. Our proposed DSNC can be simply embedded on application layer. In the practical point of view, DSNC can take a significant step towards realistic deployment integrated into WMSNs.
- We derive the closed-form equations of decoding probability that are validated by various simulations. We evaluate the effectiveness in terms of three performance metrics: decoding probability, redundancy, and image quality measurement using peak signal-to-noise ratio (PSNR). The experimental results demonstrate that the performance of the proposed DSNC outperforms the existing uploading schemes.

The remainder of this paper is organized as follows. Related work is described in Section 2. In Section 3, we present our proposed DSNC. Furthermore, we derive the closed-form equations of decoding probability as well as performance evaluation regarding the reliability in terms of decoding probability, redundancy, and PSNR compared to random linear network coding (RLNC) and SNC in Section 4 and Section 5, respectively. Finally, in Section 6 we conclude this paper along with future work.

## 2. Related Work

**Figure 1:**
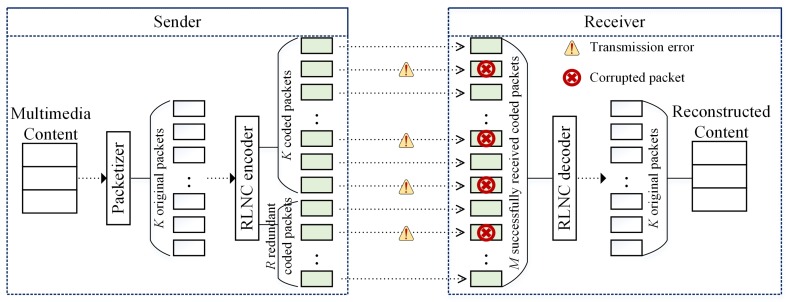
Redundancy mechanism using RLNC.

Retransmission and redundancy are two typical techniques used in WMSNs to achieve reliability [12]. Most of existing works conventionally focus on retransmission-based reliability [13]. To recover the lost packets, the sender retransmits them by getting feedback from the receiver. This retransmission-based reliability mechanism causes additional transmission overhead that not only increases network congestion but also wastes energy resource. On the other hand, redundancy-based reliability mechanism has recently attracted attention in WMSNs [14]. Since the lost packets can be recovered by utilizing some forms of coding. Among the coding techniques, RLNC was proposed to increase the reliability in transmission and solve the issue of heavy feedback signaling and retransmission [15]. Figure 1 presents RLNC encoder and decoder that are integrated on application layers of sender and receiver, respectively. The sender divides each frame into *K* packets, which are encoded using a linear combination of *K* original packets into (K+R) RLNC packets. *R* is the number of redundant packets used to increase the capability of loss recovery. The receiver can reconstruct the original packets whether the number of received packets is equal to or greater than the number of original packets. The receiver receives the transmitted network-coded packets and starts decoding as receiving a sufficient number of coded packets. The advantage of RLNC is that decoding process can complete successfully right away if the coefficient matrix achieves full rank (i.e., the coding vectors are linearly independent). The sender keeps sending the coded packets until the receiver decodes them successfully and sends an accumulated feedback to the sender. The receiver does not need to send ACK/NACK feedback for each transmission. Hence, the reliability is increased and the issue of feedback storm can be solved.

The RLNC has been exploited efficiently in many applications, such as reliable point-to-point communication [16] and efficient multicast [17,18] to enhance reliability and bandwidth efficiency. Furthermore, performance could be significantly enhanced by combining resource allocation frameworks beside the ordinary advantages of RLNC [19]. Authors in [19] showed that RLNC improved communication reliability and derived accurate closed-form equations for the probability of recovering a predetermined set of consecutive message layers. Nevertheless, none of all the above aforementioned studies investigated application on multimedia content uploading. Recently, Moritz et al. addressed the upward trend on sharing multimedia content by proposing a linear network coding approach for uplink network-coded cooperative communication with downlink energy transfer [20]. The authors derived a closed-form approximation for the system outage probability. The deployment of RLNC for uplink distributed systems was also proposed in [21]. The authors provided the analysis on outage behavior and showed that the linear network coding approach can decrease the outage probability and frame error rate.

Although RLNC can increase the reliability without using feedback and retransmission but there are two significant drawbacks: rank deficiency and high decoding complexity that are not suitable for multimedia content because of strict time constraint. To tackle these drawbacks, SNC was proposed [22]. The transmission of SNC consists of two stages: a sender sends original packets in the first stage; upon sending out all original packets, the sender continues generating RLNC packets for transmission in the second stage. Obviously, the issue of rank deficiency can be solved since the receiver can still decode some original packets even though the receiver cannot receive a sufficient number of linearly independent packets. On the other hand, SNC can achieve the same performance as the receiver receives a sufficient number of transmitted packets [22,23]. In addition, the second drawback (i.e., high decoding complexity) can be solved by brief propagation (BP) and Gaussian Elimination (GE) algorithms that are used for decoding with low computational complexity compared to RLNC [24]. However, the mentioned studies only deployed the RLNC and SNC for one-hop data uploading. Our work is the first work investigating the DSNC to enhance the efficient multimedia content uploading over WMSNs. Furthermore, we derive a closed-form equation of decoding probability which provides a theoretical analysis for further understanding of the DSNC.

## 3. Proposed DSNC

**Figure 2:**
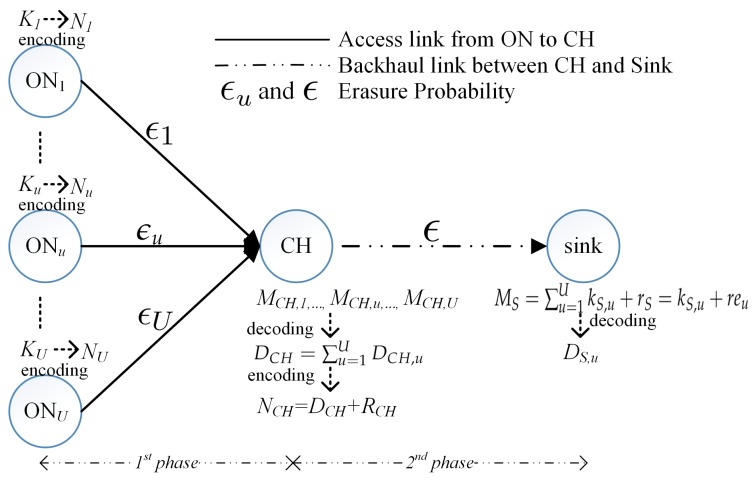
Encoding and decoding operations at ON, CH, and sink.

In this section, we present our proposed DSNC performed at each cluster in WMSNs. Figure 2 presents the encoding/decoding operations at ON, CH, and sink in an uploading scenario with multiple ONs. The wireless link from an ON to a CH is a short-ranged contention-based wireless link (e.g., WiFi) and the wireless link from the CH to a sink is a long-ranged cellular link (e.g., 4G-LTE). *U* ONs upload their own multimedia content with strict time constraint and predefined level of QoS (i.e., the data received after time constraint is useless) to a sink through a CH. Since multiple ONs upload their own content to a sink through a CH and ON cannot transmit directly to the sink, the uploading mechanism consists of two phases: (1) multiple ONs encode original packets into innovative SNC packets and transmit them to the CH using different frequencies [11]; (2) instead of forwarding all the received SNC packets from multiple senders to the sink, the CH decodes the received SNC packets and then encodes them into innovative SNC packets in the second phase. The number of innovative SNC packets is selected using the proposed bandwidth-efficient and channel-aware error control algorithm. Suppose that received signal strength indicators (RSSIs) are exchanged among sink, CH, and ONs (i.e., end users). Based on the RSSI information, the sender can estimate a packet loss ratio (PLR) at each duration of a frame transmission [25]. For clarity, we list the commonly used notations in Table 1.

### 3.1. Multimedia Content Encoding at Multiple ONs in the First Phase

**Figure 3:**
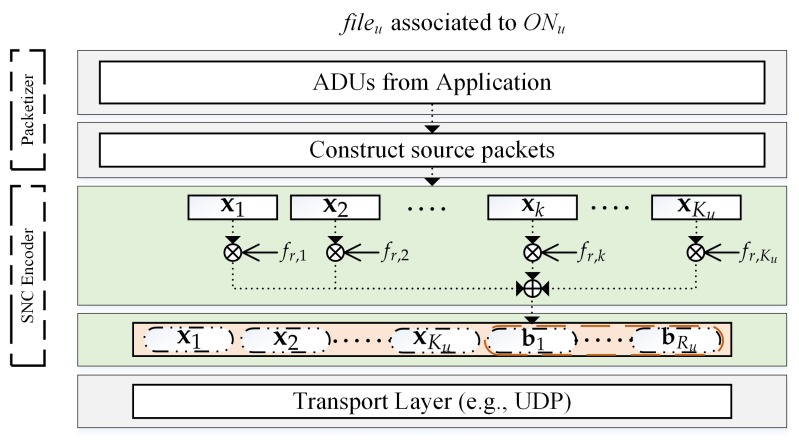
A proposed *u*th ON’s architecture.

Figure 3 presents a proposed ON’s architecture that encodes multimedia content for uploading to CH in the first phase. Let $file_{u}$ be the multimedia content (i.e., application data units (ADUs)) of the *u*th ON, where each file is partitioned into multiple packets and delivered to the SNC encoder. SNC integrates SNC encoder/decoder on application layer. Based on the transmission requirements corresponding to RSSI feedback from physical layer (PHY), the SNC encoder divides the ADU into $K_{u}$ equal-length source packets which can be presented as a vector of $K_{u}$ elements:

(1) $$X_{u}={\left[x_{1}\;\;x_{2}\;\;\cdots \;\;x_{K_{u}}\right]}^{T},$$

where ${\left(\cdot \right)}^{T}$ denotes the transpose operation. $N_{u}$ is the number of packets used for transmission from the *u*th ON to CH and $N_{u}\geq K_{u}$.

**Encoding process**: SNC encoder encodes the $K_{u}$ original source packets into $N_{u}$ SNC packets for transmission, which consist of $K_{u}$ original packets and $R_{u}$ RLNC packets (i.e., $N_{u}=K_{u}+R_{u}$). Let $b_{r}$ be an RLNC packet which is presented as follows:

(2) $$b_{r}=f_{r}X_{u}=\sum_{k=1}^{K_{u}}f_{r,k}x_{k},$$

where $f_{r,k}$ is a coefficient number of the *k*th packet of the *r*th coded packet. Each coefficient number is generated randomly over Galois Field F(q) of size *q*. We use random number generator (RNG) to generate these coefficients. Each RNG seed of the first encoded packet is transmitted in such a way that CH also can generate these same coefficients for decoding [17,22]. Lastly, $N_{u}$ SNC packets (i.e., $K_{u}$ original and $R_{u}=N_{u}-K_{u}$ RLNC packets) are delivered to transport layer based on UDP protocol for transmission. The determination of $N_{u}$ is based on RSSI feedback along with physical transmission requirements. The simple existing method is to use Equation (3) that the determination of redundant packets is equal to the expected lost packets based on erasure probability, $\epsilon_{u}$, of the channel link [26], hence a receiver can expectedly achieve the full rank of a coefficient matrix and decode all original packets.

(3) $$R_{u}=\left\lceil \frac{\epsilon_{u}K_{u}}{1-\epsilon_{u}}\right\rceil .$$

Nevertheless, this method cannot guarantee the predefined level of QoS in WMSNs as the channel condition is getting worse (i.e., proved in Section 5.2). To enhance bandwidth-efficient utilization under a predefined level of QoS, we propose a bandwidth-efficient and channel-aware error control algorithm presented in Algorithm 1 to select the optimal number of redundant packets. Our proposed Algorithm 1 is based on our analysis of decoding probability presented in Section 4. Firstly, we formulate the redundancy assignment problem as follows:

(4) $$minimize:\;\;R_{u}$$

subject to:

(5) $$\rho_{u}\geq \rho_{u,TH},$$

(6) $$R_{u}\leq R_{u,max},$$

where $\rho_{u,TH}$ is a threshold of decoding probability, constraint (5) guarantees a predefined level of QoS; $R_{u,max}$ is the maximum number of redundant packets, constraint (6) guarantees the sender cannot exceed the maximum sending rate constraint. Secondly, to solve this problem with low complexity, we perform a proximity search of the given point determined by Equation (3). Algorithm 1 starts by the given point and determines the expected decoding probability using Equations (8) and (13) from our analysis (lines 2–3). There are two cases: constraint (5) is satisfied or unsatisfied. In the first case, if constraint (5) is satisfied, it means the given point of redundancy uses more resource than the predefined quality. Hence, the algorithm reduces the number of redundant packets by one and checks the constraint (5) again. The process keeps decreasing the redundancy while constraint (5) is satisfied. The optimal value is the latest proximity value (lines 7–14). In the second case, if constraint (5) is not satisfied, it means the given point of redundancy cannot satisfy the predefined level of QoS. Hence, the algorithm increases the number of redundant packets by one until constraint (5) is satisfied (lines 15–22). Now we discuss the complexity of our proposed algorithm. The time complexity of Algorithm 1 is $O(R_{u,max})$. However, the particular time complexity is either $O(R_{u})$ (i.e., the first case) or $O(R_{u,max}-R_{u})$ (i.e., the second case) that depends on the available bandwidth, channel condition and the number of original packets. To give the further estimation of complexity, suppose that the number of original packets is $K_{u}=50$, error probability is $\epsilon_{u}=0.1$, and the available bandwidth of possibly transmitting redundant packets is $R_{u,max}=10$. The given point of searching starts from $R_{u}=6$. Hence the complexity of the Algorithm 1 is O(6) that is negligible. Hence, the propose algorithm is capable of implementing on practical sensor nodes.

**Algorithm 1:** Proposed bandwidth-efficient and channel-aware error control algorithm.**Input:** Given $K_{u}$, $\epsilon_{u}$, $\rho_{u,TH}$, $R_{u,max}$ **Output:**
$R_{u}$ 1:**procedure** 2:    $R_{u}\leftarrow$ Equation (3) 3:    $E(\rho_{u})\leftarrow$ Equations (8) and (13) 4:    **if** $E\left(\rho_{u}\right)=\rho_{u,TH}$ **then** 5:       **return** $R_{u}$ 6:    **end if** 7:    **if** $E\left(\rho_{u}\right)>\rho_{u,TH}$ **then** 8:        **for** $R_{u}-1\to 0$ **do** 9:           $E(\rho_{u})\leftarrow$ Equations (8) and (13)10:           **if** $E\left(\rho_{u}\right)<\rho_{u,TH}$ **then**11:              **return** $R_{u}+1$12:           **end if**13:        **end for**14:    **end if**15:    **if** $E\left(\rho_{u}\right)<\rho_{u,TH}$ **then**16:        **for** $R_{u}+1\to R_{u,max}$ **do**17:           $E(\rho_{u})\leftarrow$ Equations (8) and (13)18:           **if** $E\left(\rho_{u}\right)>\rho_{u,TH}$ **then**19:              **return** $R_{u}$20:           **end if**21:        **end for**22:    **end if**23:**end procedure**

**Decoding process**: the received packets can be presented as follows:

(7) $$Y_{u}=H_{u}C_{u}X_{u},$$

where (i) $H_{u}$ is an $N_{u}\times N_{u}$ diagonal transfer matrix from the *u*th ON to CH, where the diagonal component is one with the probability of $1-\epsilon_{u}$ and zeros with the probability of $\epsilon_{u}$ (i.e., erasure probability); (ii) $C_{u}=\left[I_{K_{u}};F_{u}\right]$ is an $N_{u}\times K_{u}$ coefficient matrix over the Galois field $F_{≊}q^{N_{u}\times K_{u}}$ that is vertically concatenated from the identity matrix of size $K_{u}$ (i.e., transmission of original packets) and a coefficient matrix used to encode the RLNC packets (i.e., $F_{u}\in F_{≊}q^{R_{u}\times K_{u}}$); (iii) $X_{u}$ is a matrix representing the original packets; (iv) $Y_{u}$ is a matrix representing the received packets. An example of coefficient matrix $C_{u}$ is shown in Figure 5a. The CH can decode successfully all $K_{u}$ source packets as the coefficient coding vectors of the received packets achieves the full rank of $K_{u}$ (i.e., $rank\left(H_{u}C_{u}\right)=K_{u}$). Otherwise, the CH can also decode a fraction of $K_{u}$ source packets if the uncoded packets are decoded successfully. The SNC decoder uses BP and GE algorithms for decoding with low computational complexity compared to RLNC presented in [24].

### 3.2. DSNC Execution at CH in the Second Phase

In this subsection, we present our proposed DSNC implemented at CH. Instead of forwarding all the received SNC packets from multiple senders to the sink, the CH performs DSNC in the second phase. Sink can simply use SNC decoder to decode all the original packets before the packet ordering for each individual ON.

**Figure 4:**
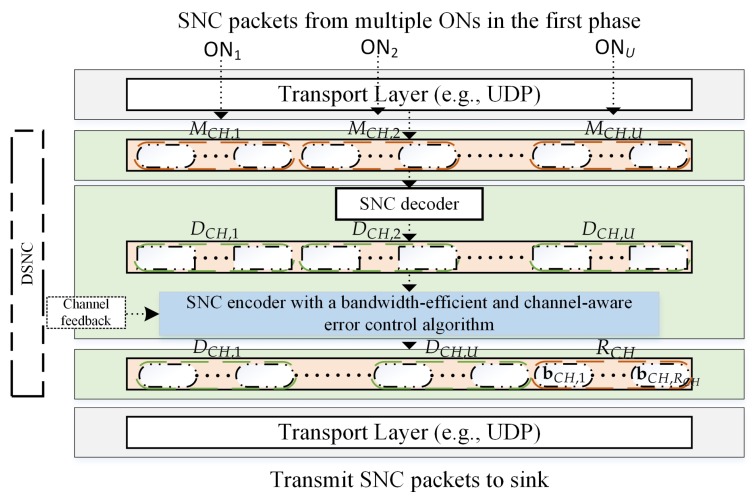
Proposed DSNC solution for multimedia uploading.

**DSNC at CH**: Figure 4 presents our proposed DSNC implemented at CH. Our proposal is integrated on application layer with the existing PHY transmission procedure. The transport layer sends the received SNC packets of each ON to SNC decoder, which decodes all received SNC packets from multiple ONs. Please note that $D_{CH,u}$ and $D_{S,u}$ are the numbers of the successfully decoded SNC packets of the *u*th ON at CH and sink, respectively, and they meet the condition $K_{u}\geq D_{CH,u}\geq D_{S,u}$. All these successfully decoded packets $D_{CH}=\sum_{u=1}^{U}D_{CH,u}$ at CH are encoded into $N_{CH}=D_{CH}+R_{CH}$ innovative SNC packets using SNC encoder, where $D_{CH}$ and $R_{CH}$ be the numbers of successfully decoded packets and RLNC packets, respectively. The $R_{CH}$ is determined using Algorithm 1 with a minor modification. We change the input information such as $K_{u}$ to $D_{CH}$, $\epsilon_{u}$ to ϵ, $\rho_{u,TH}$ to $\rho_{CH,TH}$, and $R_{u,max}$ to $R_{CH,max}$ (i.e., the maximum number of RLNC packets that the CH can transmit in the second phase). Then all of those innovative SNC packets are used for transmission to the sink. The time complexity for multiple sources of our proposed scheme at CH will be $O(R_{CH,max})$. Since the complexity only depends on the maximum total number of redundant packets used at CH which is associated with the number of ONs. As the number of ONs increases, the more redundant packets are used leading to the time complexity also increases.

**Decoding process at sink**: Clearly, the encoded packet in the second phase is a linear combination of all successfully decoded packets from all ONs. Hence, the sink cannot decode each data flow separately. To decode each data flow from each ON, the sink simply uses the BP and GE algorithms [24] similar to SNC to decode all data from ONs, and then performing the packet reordering to separate each data flow.

The DSNC can increase efficiently the multimedia uploading because of two advantages: (1) in the access link side (i.e., in the first phase from multiple ONs to CH): ON can upload a fraction of data even if the measured RSSI used for physical uplink scheduling is not accurate. This issue causes transmission error and often happens in practical wireless networks due to measurement error and the mobility of ONs [25]. On the other hand, ON can upload all data easily without using feedback and retransmission since CH can decode all transmitted packets as receiving a sufficient number of SNC packets; (2) in the backhaul link side (i.e., in the second phase from CH to sink): the reliability performance can be achieved by the substantial improvement since the generation size used for RLNC encoding increases leading to the increase in reliability [15]. As seen, the reliability improvement comes from the cost of computational complexity. However, this issue can be easily solved by the powerful computational capability of the current smart sink [27].

## 4. Performance Analysis

In this section, we derive the closed-form equations for decoding probability of our proposed DSNC. This performance metric is widely used to evaluate the reliability in transmission. The decoding probability $\rho_{S,u}$ of each ON at sink is defined as the ratio of the number of the successfully decoded packets $D_{S,u}$ at destination (i.e., sink) over $K_{u}$ source packets. The expected value of decoding probability is defined as follows:

(8) $$E\left(\rho_{S,u}\right)=\frac{E(D_{S,u})}{K_{u}}=\frac{\sum_{d_{S,u}=1}^{K_{u}}d_{S,u}\mathrm{Pr}\left(D_{S,u}=d_{S,u}\right)}{K_{u}}.$$

Our objective is to derive the probability $\mathrm{Pr}(D_{S,u}=d_{S,u})$ of the *u*th ON at sink, note that $D_{S,u}\leq K_{u}$ because of erasure channel and $d_{S,u}$ is a sliding variable of the successfully decoded number of packets. Performance analysis of SNC can be treated as the uploading scenario with a single ON since CH treats each data flow individually. For simplicity, we start the analysis with a single ON over one-hop transmission, two-hop transmission, and extending to multiple ONs of our proposed DSNC.

### 4.1. A Single ON over One-Hop Transmission

First, we describe the decoding probability of SNC over one-hop transmission with a single ON${}_{u}$ and CH in the first phase derived in [28]. Sender encodes $K_{u}$ original packets into $N_{u}$ SNC packets for transmission. Please note that $N_{u}=K_{u}+R_{u}$ consists of $K_{u}$ uncoded packets and $R_{u}$ RLNC packets. The probability **Pr**$\left(M_{CH,u},N_{u}\right)$ that the CH receives $M_{CH,u}$ (i.e., the number of received packets) over $N_{u}$ transmissions that follows a binomial distribution [15] with the erasure probability $\epsilon_{u}$ of the access link.

(9) Pr(MCH,u,Nu)=NuMCH,u(1−ϵu)MCH,uϵu(Nu−MCH,u).

The decoding capability at CH can be classified into two cases: decoding a fraction of source data if $D_{CH,u}<K_{u}$ and decoding all $K_{u}$ original packets if $D_{CH,u}=K_{u}$, note that $D_{CH,u}$ is the number of the successfully decoded packets at CH and $K_{u}$ is the number of the original packets used for transmission in the first phase. Let $k_{CH,u}$ and $r_{CH,u}$ be the numbers of uncoded (i.e., original) packets, and encoded packets (i.e., RLNC packets) of ON${}_{u}$ that are received at the CH, respectively. The random variables of $k_{CH,u}$ and $r_{CH,u}$ also follow the binomial distributions $\mathrm{Pr}(k_{CH,u},K_{u})$ and $\mathrm{Pr}(r_{CH,u},R_{u})$ independently since the channel is memoryless.

**Figure 5:**
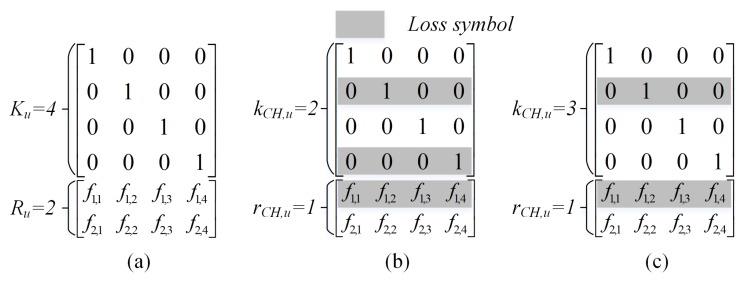
Representative matrix of coefficient coding information at ON and CH; (**a**) ON${}_{u}$; (**b**) case of $D_{CH,u}=2<K_{u}=4$; (**c**) case of $D_{CH,u}=K_{u}=4$.

Figure 5 presents an illustration of two possible decoding cases by a coefficient coding matrix. Assume that the number of original packets (i.e., uncoded packets) is $K_{u}=4$ and the number of RLNC packets is $R_{u}=2$ (i.e., Figure 5a). In the case of $D_{CH,u}<K_{u}$ shown in Figure 5b, the CH receives three packets but only decodes successfully two decoded original packets. Since the coefficient coding matrix needs to achieve the rank of 4 but the rank is only 3. Hence, one successfully received RLNC packet becomes useless. In the case of $D_{CH,u}=K_{u}$ shown in Figure 5c, the CH can decode successfully all 4 packets since the coefficient coding matrix achieves the rank of 4. Now we describe the analysis of two cases in detail:

- In the case of $D_{CH,u}<K_{u}$ (i.e., example shown in Figure 5b): CH only can decode successfully $k_{CH,u}$ uncoded packets (i.e., $D_{CH,u}=k_{CH,u}$) and the coefficient coding vectors of all the received packets cannot achieve the rank of $K_{u}$.
  (10) $$\mathrm{Pr}\left(D_{CH,u}=k_{CH,u}\right)=\mathrm{Pr}\left(k_{CH,u},K_{u}\right)\left(1-\sum_{r_{CH,u}=K_{u}-k_{CH,u}}^{R_{u}}\mathrm{Pr}\left(r_{CH,u},R_{u}\right)f_{K_{u}}\left(K_{u}-k_{CH,u},r_{CH,u}\right)\right),$$
  where $R_{u}=N_{u}-K_{u}$ is the number of RLNC packets used for transmission, in which $K_{u}$ and $R_{u}$ represent the ranks of coefficient matrix and redundant packets, respectively; $f_{K_{u}}\left(K_{u}-k_{CH,u},r_{CH,u}\right)$ is the probability that the $r_{CH,u}\times K_{u}$ coefficient coding matrix achieves the rank of $\left(K_{u}-k_{CH,u}\right)$ derived in [22].
  (11) $$f_{K_{u}}\left(K_{u}-k_{CH,u},r_{CH,u}\right)=\prod_{j=0}^{K_{u}-k_{CH,u}-1}\left(1-\frac{1}{q^{r_{CH,u}-j}}\right).$$
- In the case of $D_{CH,u}=K_{u}$ (i.e., example shown in Figure 5c): obviously, CH can decode all $K_{u}$ source packets as the matrix of received coefficient coding vectors achieves the rank of $K_{u}$. The $\mathrm{Pr}(D_{CH,u}=K_{u})$ can be simply expressed as follows:
  (12) $$\mathrm{Pr}\left(D_{CH,u}=K_{u}\right)=\sum_{k_{CH,u}=0}^{K_{u}}\mathrm{Pr}\left(k_{CH,u},K_{u}\right)\sum_{r_{CH,u}=K_{u}-k_{CH,u}}^{R_{u}}\mathrm{Pr}\left(r_{CH,u},R_{u}\right)f_{K_{u}}\left(K_{u}-k_{CH,u},r_{CH,u}\right).$$
  Finally, the $\mathrm{Pr}(D_{CH,u}=d_{CH,u})$ for a single-hop wireless transmission can be derived as follows:
  (13) $$\begin{array}{c} \mathrm{Pr}\left(D_{CH,u}=d_{CH,u}\right)=\left\{\begin{array}{cc} \mathrm{Equation}\;(10) & \mathrm{if}\;d_{CH,u}<K_{u}\\ \mathrm{Equation}\;(12) & \mathrm{if}\;d_{CH,u}=K_{u} \end{array}\right.. \end{array}$$

### 4.2. A Single ON over Two-Hop Transmission

Now, we extend our analysis for the second transmission from CH to sink. The closed-form expectation of decoding probability of ON${}_{u}$ is presented as follows:

(14) $$E\left(\rho_{S,u}\right)=\frac{\sum_{d_{S,u}=1}^{K_{u}}d_{S,u}\sum_{d_{CH,u}=d_{S,u}}^{K_{u}}\mathrm{Pr}\left(D_{CH,u}=d_{CH,u}\right)\mathrm{Pr}\left(D_{S,u}=d_{S,u}|D_{CH,u}\right)}{K_{u}},$$

Since the $\mathrm{Pr}(D_{S,u}=d_{S,u})$ at the sink is the marginal distribution computed from the joint distribution $\mathrm{Pr}(D_{CH,u},D_{S,u})$. Hence, the $\mathrm{Pr}(D_{S,u}=d_{S,u})$ is expressed as follows:

$$\mathrm{Pr}\left(D_{S,u}=d_{S,u}\right)=\sum_{d_{CH,u}=d_{S,u}}^{K_{u}}\mathrm{Pr}\left(D_{CH,u}=d_{CH,u}\right)\mathrm{Pr}\left(D_{S,u}=d_{S,u}|D_{CH,u}\right),$$

where $\mathrm{Pr}(D_{CH,u}=d_{CH,u})$ is the probability of decoding successfully $d_{CH,u}$ at the CH in the first phase, which is determined using Equation (13). Please note that the sink only uses $D_{CH,u}$ decoded packets for encoding into $N_{CH,u}$ SNC packets. The conditional probability $\mathrm{Pr}(D_{S,u}=d_{S,u}|D_{CH,u})$ is the decoding probability that the sink can decode $d_{S,u}$ packets given $D_{CH,u}$. The complete closed-form equation can be defined as follows:

(15) $$\mathrm{Pr}\left(D_{S,u}=d_{S,u}|D_{CH,u}\right)=\left\{\begin{array}{c} \mathrm{Pr}\left(d_{S,u},D_{CH,u}\right)\left(1-\sum_{r_{S,u}=D_{CH,u}-d_{S,u}}^{R_{CH,u}}\mathrm{Pr}\left(r_{S,u},R_{CH,u}\right)f_{D_{CH,u}}\left(D_{CH,u}-d_{S,u},r_{S,u}\right)\right)\\ \;\mathrm{if}\;d_{S,u}<D_{CH,u}\\ \sum_{k_{S,u}=0}^{D_{CH,u}}\mathrm{Pr}\left(k_{S,u},D_{CH,u}\right)\sum_{r_{S,u}=D_{CH,u}-k_{S,u}}^{R_{CH,u}}\mathrm{Pr}\left(r_{S,u},R_{CH,u}\right)f_{D_{CH,u}}\left(D_{CH,u}-k_{S,u},r_{S,u}\right)\\ \;\mathrm{if}\;d_{S,u}=D_{CH,u} \end{array}\right.,$$

where $R_{CH,u}=N_{CH,u}-D_{CH,u}$ is the number of RLNC packets due to a single ON only; $k_{S,u}$ and $r_{S,u}$ be the numbers of uncoded packets and encoded packets received at the sink from the CH, respectively.

### 4.3. Analysis of DSNC with Multiple Source Senders

Based on the above analysis, we extent to the case of our proposed DSNC with multiple source senders. Instead of simply treating each data flow individually, CH encodes all successfully decoded packets from source senders (i.e., all ONs) in the first phase into innovative SNC packets and transmits them to the sink in the second phase.

**Figure 6:**
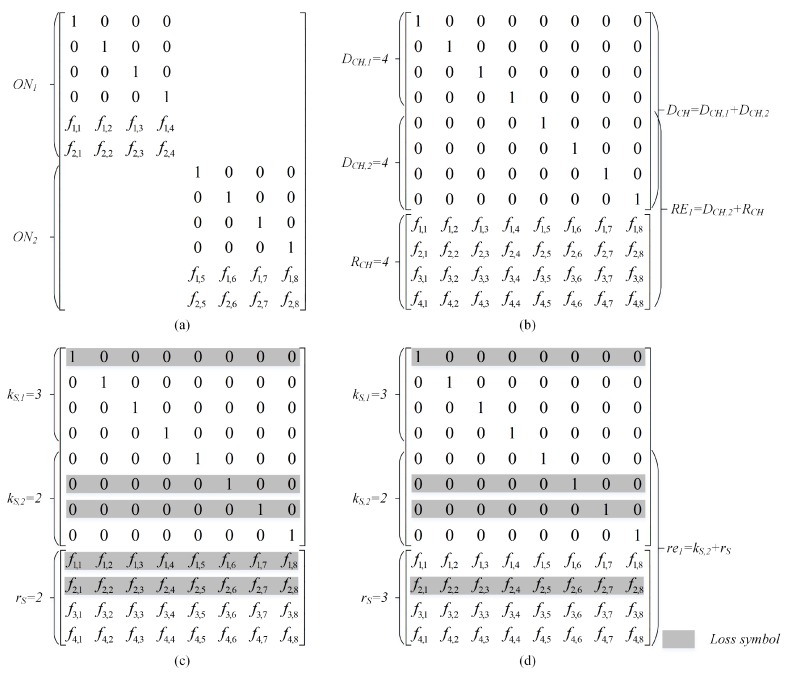
Representative matrix of coefficient coding information at CH and sink; (**a**) SNC; (**b**) DSNC; (**c**) case of $D_{S,1}=3\leq D_{CH,1}$; (**d**) case of $D_{S,1}=D_{CH,1}=4$ or $M_{S}=8$.

We firstly extend the analysis to the case of two ONs. Please note that CH uses all successfully decoded packets from both source senders for encoding. Hence, the total number of SNC packets used for transmission from the CH to sink is determined as $N_{CH}=D_{CH,1}+D_{CH,2}+R_{CH}$. Figure 6a,b present the coefficient coding matrices at CH performed SNC individually and DSNCs, respectively. For simplicity, assuming that CH can decode successfully all four transmitted packets from both ONs in the illustration. Obviously, each data flow can be decoded as the coefficient coding matrix achieves the rank of $D_{CH}=\sum_{u=1}^{2}D_{CH,u}$. On the other words, sink needs to receive at least $D_{CH}$ packets.

Now, we perform the derivation for ON${}_{1}$, because the case of ON${}_{2}$ can easily been analyzed in the same way. The decoding probability of ON${}_{1}$ can be derived as follows:

(16) $$\mathrm{Pr}\left(D_{S,1}=d_{S,1}\right)=\sum_{d_{CH,1}=d_{S,1}}^{K_{1}}\mathrm{Pr}\left(D_{CH,1}=d_{CH,1}\right)\sum_{d_{CH,2}=0}^{K_{2}}\mathrm{Pr}\left(D_{CH,2}=d_{CH,2}\right)\mathrm{Pr}\left(D_{S,1}=d_{S,1}|D_{CH}\right).$$

The terms of $\mathrm{Pr}(D_{CH,1}=d_{CH,1})$ and $\mathrm{Pr}(D_{CH,2}=d_{CH,2})$ can be determined using in such a similar way of Equation (13). The last term is conditional probability $\mathrm{Pr}(D_{S,1}=d_{S,1}|D_{CH}=\sum_{u=1}^{2}D_{CH,u})$ that the sink can decode the data flow of ON${}_{u}$ depending on two cases: decoding a fraction of source data if $D_{S,1}<D_{CH,1}$ and decoding all $D_{CH,1}$ if $D_{S,1}=D_{CH,1}$ or corresponding to $M_{S}=D_{CH}$. Let $k_{S,u}$ and $r_{S}$ be the numbers of received uncoded packets of the *u*th ON and received RLNC packets at sink, respectively. $M_{S}=\sum_{u=1}^{2}k_{S,u}+r_{S}$ is the number of received packets at sink.

- The case of $D_{S,1}\leq D_{CH,1}$ (i.e., example shown in Figure 6c): sink only can decode successfully $k_{S,1}$ uncoded packets (i.e., $D_{S,u}=k_{S,1}$) and the coefficient coding vectors of $k_{S,2}$ uncoded and $r_{S}$ RLNC packets are not linearly independent.
  (17) $$\mathrm{Pr}\left(D_{S,1}=d_{S,1}|D_{CH}\right)=\mathrm{Pr}\left(d_{S,1},D_{CH,1}\right)\left(1-\sum_{re_{1}=D_{CH,1}-d_{S,1}}^{RE_{1}}\mathrm{Pr}\left(re_{1},RE_{1}\right)f_{D_{CH,1}}\left(D_{CH,1}-d_{S,1},re_{1}\right)\right),$$
  where $RE_{1}=D_{CH,2}+R_{CH}$ is treated as the number of redundant packets of the 1st ON. Each ON treats the received packets of other ONs as redundancy. Examples of the $RE_{1}$ and $R_{CH}$ are presented in Figure 6b.
- The case of $D_{S,1}=D_{CH,1}$ (i.e., example shown in Figure 6d): obviously, CH can decode all $D_{CH,u}$ source packets as the matrix of received coefficient coding vectors achieves the rank of $D_{CH,u}$.
  (18) $$\mathrm{Pr}\left(D_{S,1}=D_{CH,1}|D_{CH}\right)=\sum_{d_{S,1}=0}^{D_{CH,1}}\mathrm{Pr}\left(d_{S,1},D_{CH,1}\right)\sum_{re_{1}=D_{CH,1}-d_{S,1}}^{RE_{1}}\mathrm{Pr}\left(re_{1},RE_{1}\right)f_{D_{CH,1}}\left(D_{CH,1}-d_{S,1},re_{1}\right)$$

Hence, the final closed-form equation of decoding probability of the *u*th ON at sink can be derived as follows:

(19) $$\mathrm{Pr}\left(D_{S,u}=d_{S,u}\right)=\sum_{d_{CH,u}=d_{S,u}}^{K_{u}}\mathrm{Pr}\left(D_{CH,u}=d_{CH,u}\right)\prod_{j\neq u}^{U}\sum_{d_{CH,j}=0}^{K_{j}}\mathrm{Pr}\left(D_{CH,j}=d_{CH,j}\right)\mathrm{Pr}\left(D_{S,u}=d_{S,u}|D_{CH}\right),$$

with $\mathrm{Pr}(D_{CH,u}=d_{CH,u})$ can be determined using in the similar way of Equation (13), and the conditional probability $\mathrm{Pr}(D_{S,u}=d_{S,u}|D_{CH}=\sum_{u=1}^{U}D_{CH,u})$ is presented as follows:

(20) $$\mathrm{Pr}\left(D_{S,u}=d_{S,u}|D_{CH}\right)=\left\{\begin{array}{c} \mathrm{Pr}\left(d_{S,u},D_{CH,u}\right)\left(1-\sum_{re_{u}=D_{CH,u}-d_{S,u}}^{RE_{u}}\mathrm{Pr}\left(re_{u},RE_{u}\right)f_{D_{CH,u}}\left(D_{CH,u}-d_{S,u},re_{u}\right)\right)\\ \;\mathrm{if}\;d_{S,u}<D_{CH,u}\\ \sum_{d_{S,u}=0}^{D_{CH,u}}\mathrm{Pr}\left(d_{S,u},D_{CH,u}\right)\sum_{re_{u}=D_{CH,u}-d_{S,u}}^{RE_{u}}\mathrm{Pr}\left(re_{u},RE_{u}\right)f_{D_{CH,u}}\left(D_{CH,u}-d_{S,u},re_{u}\right)\\ \;\mathrm{if}\;d_{S,u}=D_{CH,u} \end{array}\right.,$$

where $RE_{u}=\sum_{j\neq u}^{U}D_{CH,j}+R_{CH}$.

## 5. Performance Evaluation

For performance evaluation, we performed various simulations that firstly validate our theoretical analysis. The analytical and simulation results matched well with a difference of 0.05%. Secondly, we present the comparative performance analysis with the existing uploading schemes. Each simulation was run 100,000 times in the Matlab environment with a confidence interval of at least 95%. Galois field size of 8 is selected to guarantee linear independence with a very high probability at application layer [29]. There is no standard method for selecting a field size, but there are majority of works that consider the Galois field size of 8 [19,30]. Table 2 describes the main simulation parameters.

### 5.1. Validation of Theoretical Analysis

**Figure 7:**
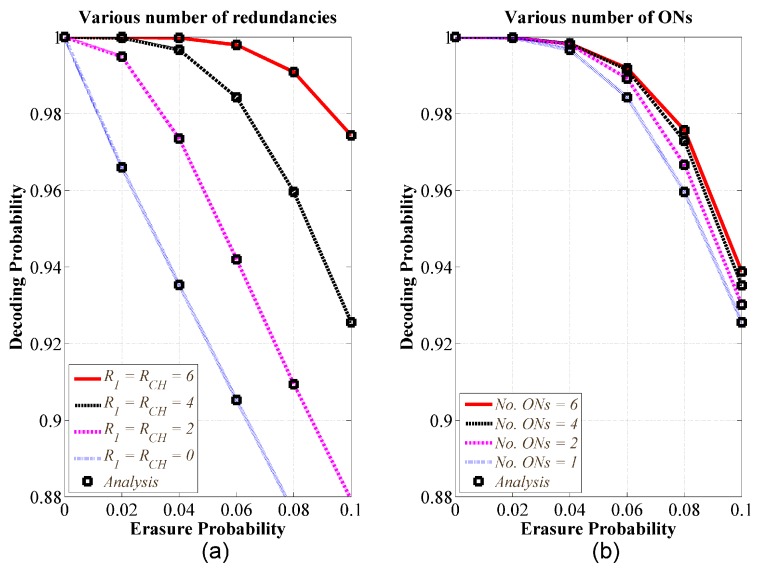
(**a**) Decoding probability versus erasure probability with various redundancies and a single ON; (**b**) Decoding probability versus erasure probability with various numbers of ONs and redundant packets of 4; K=30, and q=256.

Firstly, we validated the theoretical analysis presented in Section 4.2. In addition, we proved that the performance of decoding probability that depended on the selection of redundancy. In this evaluation, suppose that a single ON${}_{1}$ encoded $K_{1}=30$ original packets for uploading to sink through CH. Figure 7a showed the decoding probability versus erasure probability with various redundancies. We assume that two links have the same erasure probability that is widely used for two-hop communication [31]. Redundancies at ON${}_{1}$ and CH are similar. As seen, when the overhead was zero, the decoding probability was similar to that of the transmission scheme without coding. The increase in redundancy leaded to the increase in the decoding probability. When the sender increased the sufficient number of redundant packets, the receiver could achieve the maximum decoding probability.

Secondly, we validated the theoretical analysis for our proposed DSNC presented in Section 4.3. Furthermore, we evaluated the impact of the number of ONs on the performance. Figure 7b showed the decoding probability versus erasure probability with various numbers of ONs. The results were decoding probabilities of the 1st ON, where channel conditions of access link (i.e., between ON${}_{1}$ and CH) and backhaul link (i.e., between CH and sink) have the similar erasure probability. Other access links had erasure probability of zero, which means no error over other access links. We considered this configuration since we mainly focused on the performance of our proposed DSNC implemented as CH in the second phase. Each ON encoded $K_{u}=30$ original packets for uploading and used redundancy of $R_{u}=R_{CH}=4$ for transmission. The results demonstrated that the increase in the number of ONs leaded to the increase in performance of decoding probability.

**Figure 8:**
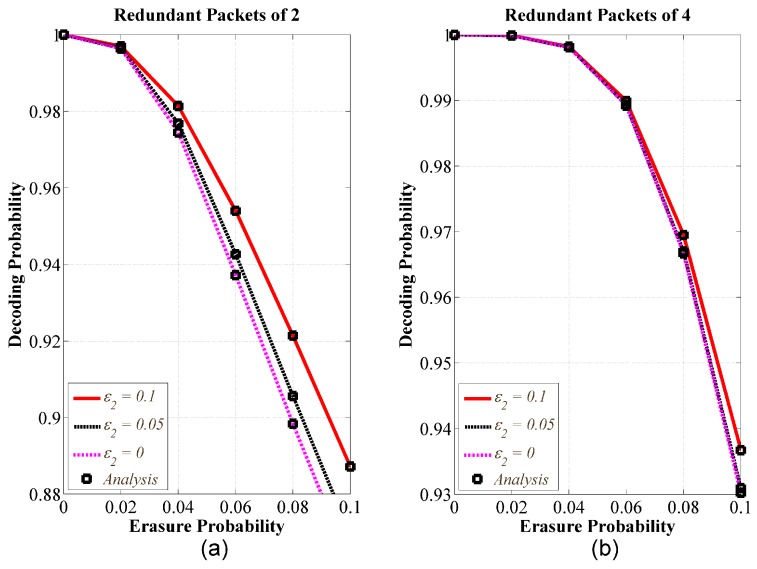
Decoding probability versus erasure probability with various erasure probabilities of the access link between ON${}_{2}$ and CH; 2 ONs, $K_{u}=30$, and q=256; (**a**) $R_{1}=R_{2}=2$, $R_{CH}=4$; (**b**) $R_{1}=R_{2}=4$, $R_{CH}=8$.

Thirdly, we evaluated the impact of erasure probabilities of the other adjacent access links. We performed simulation with two ONs, and various erasure probabilities of the access link between ON${}_{2}$ and CH (i.e., $\epsilon_{2}$). Figure 8 showed the decoding probability of ON${}_{1}$. The simulation results also matched well with our theoretical analysis. Figure 8a,b are results of decoding probability corresponding to $R_{1}=R_{2}=2$ and $R_{1}=R_{2}=4$, respectively. Figure 8a showed that given the channel condition of access link 1 and the number of predefined redundant packets, the performance of decoding probability increases as the channel condition of the 2nd access link increases. In addition, Figure 8b demonstrated that if the number of predefined redundant packets is assigned sufficient enough. The impact of erasure probability of the $\epsilon_{2}$ is trivial.

### 5.2. Comparative Performance Analysis

In this subsection, we compared the performance of our scheme to two other baseline schemes that were RLNC [16] and SNC [23]. This is because both of them employed redundancy-based reliability mechanism to use the advantage of coding for providing the capability of error correction over lossy wireless channel like our proposal scheme. We performed simulations with two ONs uploading to sink through CH. Each ON also encoded $K_{u}=30$ original packets for uploading. Each packet was 1500 bytes and transmission rate for each ON’s link was 480 Kbps (i.e., maximum sending rate was 40 packets per second). We evaluate the performance of the 1st ON. Performance metrics used for comparative analysis were decoding probability, redundancy, and PSNR for image quality measurement. Channel conditions of access link (i.e., between ON${}_{1}$ and CH) and backhaul link (i.e., between CH and sink) have the similar erasure probability. Other access link (i.e., between ON${}_{2}$ and CH) has erasure probability of zero, which means no error over this access link.

**Figure 9:**
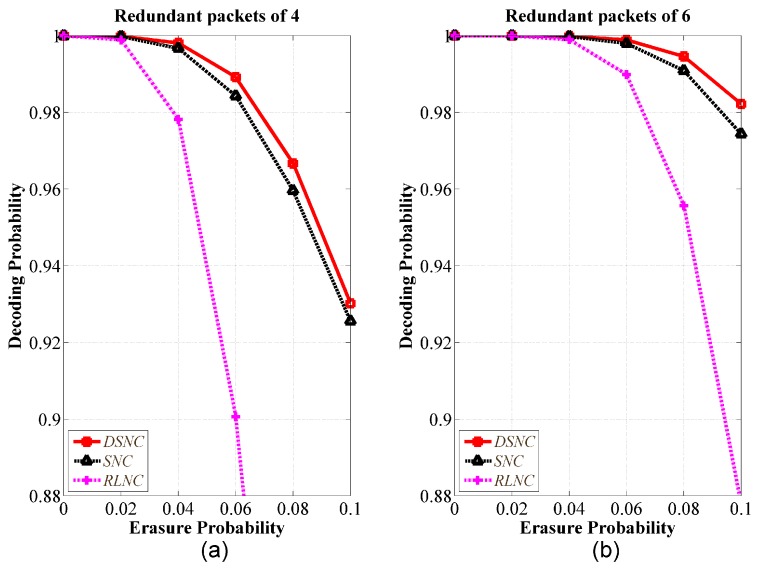
Performance comparison of decoding probability versus erasure probability with predefined redundancies; 2 ONs, $K_{u}=30$, and q=256; (**a**) the number of redundant packets is 4; (**b**) the number of redundant packets is 6.

Figure 9 presented the performance of decoding probability of the 1st ON versus erasure probability with various redundancies compared to RLNC and SNC. We did not apply the method to dynamically determine redundancy according to channel condition, since we focused on the evaluation of the general impact of redundancy to all schemes. Given channel condition and predetermined redundancy, the DSNC showed better performance than SNC and RLNC. We selected the same numbers of redundant packets as 4 and 6, leading to the results shown in Figure 9a,b, respectively. As channel condition was getting worse, the DSNC showed better performance since the RLNC had rank deficiency disadvantage. Please note that we performed simulation with two ONs, but as shown in Figure 7b, our proposed scheme could achieve better performance as the number of ONs increases.

**Figure 10:**
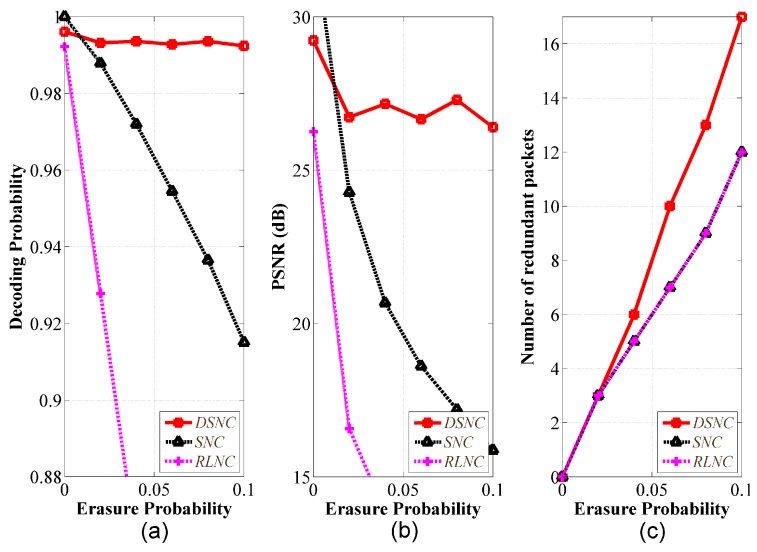
Performance comparison with adaptive method for redundancy with Lena image and size of 501 KB; (**a**) decoding probability; (**b**) PSNR; (**c**) number of redundant packet; generation size of 100, q=256.

Lastly, we used Lena image for transmission and evaluated with the adaptive method to dynamically determine redundancy according to channel condition. The image file is still large for WMSNs. We divided the file into multiple segments of 100 packets. The predefined level of QoS was the decoding probability threshold of 0.99. Figure 10a–c presented the performance of decoding probability, PSNR, and redundancy, respectively. This simulation differs from the results in Figure 9, because we applied the adaptive method to dynamically determine redundancy according to channel condition in order to show the advantage of the proposed bandwidth-efficient and channel-aware error control algorithm. Other schemes use the existing method shown in Equation (3). As shown in Figure 10a,b, the DSNC could still satisfy the predefined level of QoS even the channel condition getting worse. Especially, the performance gap increases as the channel condition is getting worse. Accordingly, Figure 10c shows that the DSNC could adaptively determine the redundancy better than the other schemes to achieve the decoding probability threshold.

## 6. Conclusions

We proposed a distributed systematic network coding called DSNC for multimedia content uploading over WMSNs. In addition, we derived the closed-form equation for decoding probability analysis. Based on the analysis, we proposed a bandwidth-efficient and channel-aware error control algorithm to enhance the bandwidth-efficient utilization by dynamically determining the optimal number of innovative coded packets. The experiment results verified our mathematical equations and demonstrated that the proposed distributed systematic network coding outperformed the random linear network coding-based scheme and systematic network coding-based scheme in terms of decoding probability, redundancy, and image quality measurement. For future research, we extend our work to the vehicular sensor networks for multimedia collection and dissemination, in which the mobility challenge is taken under consideration.

## Figures and Tables

**Table 1:** Commonly used notation.

Notation	Definition
*U*	The number of ONs
$K_{u}$	The number of original packets used for a frame transmission of the *u*th ON
$R_{u}$	The number of RLNC packets of the *u*th ON used for a frame transmission
$R_{u,max}$	The maximum number of RLNC packets of the *u*th ON used for transmission
$N_{u}$	The total number of SNC packets used for transmission of the *u*th ON, note that $N_{u}=K_{u}+R_{u}$
$M_{CH,u}$	The total number of received SNC packets of the *u*th ON at CH, note that $M_{CH,u}=k_{CH,u}+r_{CH,u}$
$k_{CH,u}$	The number of received uncoded packets of the *u*th ON at CH
$r_{CH,u}$	The number of received RLNC packets of the *u*th ON at CH
$D_{CH,u}$	The number of successfully decoded packets of the *u*th ON at CH
$D_{CH}$	The total number of successfully decoded packets at CH, note that $D_{CH}=\sum_{u=1}^{U}D_{CH,u}$
$R_{CH,u}$	The number of RLNC packets used for transmission of the *u*th ON from CH to sink
$R_{CH}$	The total number of RLNC packets used for transmission from CH to sink, note that $R_{CH}=\sum_{u=1}^{U}R_{CH,u}$
$N_{CH,u}$	The number of SNC packets used for transmission of the *u*th ON from CH to sink, note that $N_{CH,u}=D_{CH,u}+R_{CH,u}$
$N_{CH}$	The total number of SNC packets used for transmission from CH to sink, note that $N_{CH}=\sum_{u=1}^{U}N_{CH,u}$
$M_{S}$	The total number of received packets at sink
$D_{S,u}$	The number of successfully decoded packets of the *u*th ON at sink
$k_{S,u}$	The number of received uncoded packets of the *u*th ON at sink
$r_{S}$	The total number of received RLNC packets at sink
$\rho_{S,u}$	Decoding probability of the *u*th ON at sink
$\rho_{u,TH}$	Decoding probability threshold of the *u*th ON
$E(\rho_{S,u})$	Expected decoding probability of the *u*th ON at sink
$\epsilon_{u}$	Erasure probability of access link from the *u*th ON to CH
ϵ	Erasure probability of the backhaul link from CH to sink

**Table 2:** Simulation parameters.

Parameter	Value
Galois Field size *q*	256
Generation size $K_{u}$	30
Packet size	1500 bytes
Transmission rate	480 Kbps
Image used for transmission	Lena
Size of Lena Image	501 KB
Generation size for image transmission	100

## Abbreviations

The following abbreviations are used in this manuscript:

ADUs	Application data units
BP	Brief propagation
CH	Cluster head
DSNC	Distributed systematic network coding
GE	Gaussian Elimination
ON	Ordinary node
PHY	Physical layer
PLR	Packet loss ratio
QoS	Quality of service
RLNC	Random linear network coding
RSSI	Received Signal Strength Indicator
SNC	Systematic network coding
WMSNs	Wireless multimedia sensor networks
